# Supplementary material for: Cognitive processes during deception about attitudes revisited: a replication study
Source: Soc Cogn Affect Neurosci. 2020 Aug 20;15(8):839–48. doi: 10.1093/scan/nsaa107 (PMC7543939; doi:10.1093/scan/nsaa107)
Supplement: nsaa107_Supp [file nsaa107_supp.zip › nsaa107_Supp/scan-20-041-File001.docx]

Cognitive processes during deception about attitudes revisited:

A replication study

Vera Scheuble & André Beauducel

Institute of Psychology, University of Bonn, Kaiser-Karl-Ring 9, 53111 Bonn, Germany

Corresponding author:

Vera Scheuble

Institute of Psychology

University of Bonn

Kaiser-Karl-Ring 9

D-53111 Bonn

# Abstract

ERP studies about deception often apply recognition tasks. It remains questionable whether reported ERP patterns and cognitive processes can be generalized to other contexts. As the study by Johnson *et al.* (2008) fills this gap by investigating deception regarding attitudes, we tried to replicate it. Participants (*N*=99) were instructed to lie or tell the truth about their attitudes. We obtained the same results as Johnson *et al.* (2008): Lies were accompanied by enhanced medial frontal negativities (MFN) and suppressed late positive components (LPCs) indicating that lying relied on stronger cognitive control processes and response conflicts than being honest. The amplitudes of pre-response positivities (PRP) were reduced for lies implying that lies about attitudes were accompanied by strategic monitoring. MFN amplitudes increased and LPC amplitudes decreased for lies about positively valued items revealing that lying about positively valued items is cognitively more challenging than lying about negatively valued items. As a new finding, MFN, LPC, and PRP components were neither moderated by Machiavellianism nor by changes in the attitude ratings. The results indicate that LPC, MFN, and PRP components are reliable indicators of the cognitive processes used during deception and that it is worthwhile to investigate them in further deception contexts.

Keywords: deception; MFN; LPC; attitudes; Machiavellianism

Words: 200/200

# Introduction

## ERP components in deception studies

Recently many studies have focused on the potential of ERP components to detect deception. Most of them apply recognition tasks in a forensic context. Studies investigating deception in other contexts are scarce (Leue and Beauducel, 2019). Yet, to get to know cognitive processes underlying deception in general, it is mandatory to study deception in different contexts. One promising study investigating deception about attitudes is by Johnson *et al.* (2008). They found large effects of at least *η_p_*^2^= .63 analyzing the difference between deception and honest responses for late positive components (LPC), medial frontal negativities (MFN), and a component they named pre-response positivity (PRP). The importance of reproducible psychological studies has been emphasized (Yong, 2012; Open Science Collaboration, 2015). We aimed to replicate the research findings of Johnson *et al.* (2008) to help set a solid basis for future ERP studies about lies beyond the recognition and forensic contexts. The study was pre-registered before data collection on the Open Science Framework (link to pre-registration: https://osf.io/f6w97).

The MFN is a negative deflection occurring up to 100 ms after a response at fronto-central electrodes (Johnson *et al.*, 2008). Its neural source is supposed to be the Anterior Cingulate Cortex (ACC) or areas nearby (Gehring and Willoughby, 2004; Nieuwenhuis *et al.*, 2004). Activity of the ACC has been associated with response inhibition and monitoring of conflicting response tendencies (Aron *et al.*, 2004; Botvinick, 2007). Likewise, telling a lie relies on inhibiting the truth and formulating another answer; conflicts have to be monitored and solved (Gombos, 2006; Sip *et al.*, 2008). In ERP studies, larger MFN amplitudes occurred for deceptive compared to truthful responses (Johnson *et al.*, 2004; Kireev *et al.*, 2008; Leue *et al.*, 2012; Gibbons *et al.*, 2018; Scheuble and Beauducel, 2020). Johnson *et al.* (2004, 2008) reported increased MFN amplitudes for lies about attitudes and regarding the familiarity of items. Likewise, Kireev *et al.* (2008) found a more negative amplitude of an ERP component similar to the MFN, for deception in a card game. Conversely, Suchotzki *et al.* (2015) found larger MFNs for truthful than for deceptive responses applying the Sheffield lie test. In summary, the evidence for larger MFNs for deceptive responses in other than recognition contexts relies heavily on the study by Johnson *et al.* (2008).

Another ERP component closely related to deception is the LPC, sometimes also named P300 (Johnson *et al.*, 2003, 2004, 2005, 2008; Meijer *et al.*, 2007; Polich, 2007). Since the LPC and P300 have not been associated with invariant features and these terms have been used interchangeably in the deception literature, we apply the term LPC in the following (Polich, 2007, p. 2128; Leue and Beauducel, 2019). According to Johnson’s (1986, 1988) triarchic model LPC amplitudes are affected by stimulus probability, stimulus meaning, and how much of the transmitted stimulus information is received. The majority of deception studies rely on its feature to indicate stimulus probability. When a person recognizes an item in a series of other unknown items, it is perceived as more infrequent than the unknown items and therefore accompanied by a more positive LPC amplitude, even when the recognition of the stimulus is denied (Rosenfeld *et al.*, 2013; Leue and Beauducel, 2019). Some studies, however, studied deception that did not rely on the recognition of an item (Leue and Beauducel, 2019). When attention is drawn away from a stimulus by another task, decreased LPCs can be found, since transmission of the stimulus information is hampered (Johnson *et al.*, 1986, 1988; Palmer *et al.*, 1994; Beauducel *et al.*, 2006). Lying could be considered as such a dual-task (Johnson *et al.*, 2003, 2004, 2005, 2008). Correspondingly, in these studies suppressed LPC amplitudes occurred for deceptive compared to honest responses (Vendemia and Buzan, 2005; Dong *et al.*, 2010; Meek *et al.*, 2013; Pfister *et al.*, 2014). Johnson *et al.* (2008) observed this pattern of LPCs for lies about attitudes. Furthermore, it has been observed for lies about the evaluation of attractiveness, lies about known facts, and knowledge received during an examination session (Vendemia and Buzan, 2005; Dong *et al.*, 2010; Meek *et al.*, 2013; Pfister *et al.*, 2014). However, to improve our knowledge on cognitive processes during lies, further studies applying deception tasks in multiple contexts have to be conducted and reliable results have to be found. Replicating the results of prior studies, like those of Johnson *et al.* (2008), is a step towards this goal.

Johnson *et al.* (2004, 2005, 2008) also analyzed a response-synchronized ERP component preceding a response, which they named PRP. Decreased PRP amplitudes have been related by Johnson *et al*. (2004, 2005, 2008) to strategic monitoring and “processing required to make intention-based responses” (Johnson, 2014, p. 254). Strategic, higher-order monitoring processes ensure that a long-term goal is kept in mind and responses align with this goal. Conversely, tactical monitoring defines cognitive control processes that continue over a short period of time, like the time between a stimulus presentation and a response (Johnson *et al.*, 2004, 2005, 2008). In a previous study by Johnson *et al.* (2004, 2005) participants completed a recognition task. Tactical monitoring was required when they were instructed to lie on every trial (directed lies). In a different task, strategic monitoring was needed when participants chose on their own to lie or not, but were instructed to give in total an equal amount of deceptive and honest responses (self-generated lies). PRP amplitudes were slightly attenuated for directed lies, but highly attenuated for self-generated lies requiring strategic monitoring (Johnson *et al.*, 2004, 2005). Conversely, for directed lies about attitudes Johnson *et al.* (2008) found a decrease of PRP amplitudes similar to the decrease for self-generated lies. They concluded that lies about attitudes are accompanied by strategic monitoring. Due to the fact that the PRP component occurs before a response, it is possible that it temporally overlaps with a Contingent Negative Variation (slow negative potential between a warning stimuli and a response signal) or the Bereitschaftspotential (slow negative shift preceding a response), if either of them is present. There are, to our knowledge, no deception studies from other researchers analyzing the PRP component. Overall, this motivates the replication of this relatively new and promising ERP component of deception studies. Although Tu *et al.* (2009) also analyzed lies about attitudes it is hard to draw a conclusion about the reproducibility of the findings by Johnson *et al.* (2008) from their study because they applied a different task and analyzed stimulus locked ERP components (N400-700 and P1000-2000).

Johnson *et al.* (2008) found that lying about positively valued (agree) items was more conflicting and required more cognitive resources than lying about negatively valued (disagree) items: MFN amplitudes were enhanced and LPC amplitudes were attenuated for deceptive responses of agree items. Furthermore, PRPs were smaller for agree than for disagree items. They reasoned that lying about positively valued topics could be perceived as a denial of the self and is accordingly accompanied by more conflict. Moreover, they explained that lying about negatively rated items could be seen as a form of compliance. In everyday life, overrating the positive aspects of a negatively valued topic is probably often perceived as socially more acceptable than saying the truth. In a study by Gershoff *et al.* (2008) people overestimated the population consensus to a greater extent for items they liked than for items they disliked. Furthermore, they reported that it is easier for people to recall positive features of negatively rated items than vice versa. The importance of positive-negative asymmetries has been underlined in different domains and is predictive for judgements and related behavior (Fazio *et al.*, 2015). Since the recognition of negative aspects from positively valued topics seems to be more challenging and people rather overestimate the population consensus for themes they like, lying about positively valued items should require more cognitive control processes resulting in larger effects on ERP components for positively than negatively valued items. Even though this effect was already shown in Johnson *et al.* (2008), they did not expect effects of the valence of the items and a replication of it was especially needed.

## *Deception and Machiavellianism*

Results of different studies, and findings from deception studies in particular, imply that MFNs and LPCs can be modulated by personality traits. Individuals scoring higher in justice sensitivity showed larger differences for early LPC as well as MFN amplitudes between deceptive and honest responses in a study by Leue et al. (2012). Machiavellianism is characterized by a manipulative and strategic behavior, a cynical worldview, callous affect, and a lack of morality (Jones and Paulhus, 2014). Individuals scoring higher on Machiavellianism deceive more frequently, deceiving seems to be cognitively less strenuous for them, and they rate their abilities in deceiving higher (Kashy and DePaulo, 1996; Gozna et al., 2001; Azizli et al., 2016; Wissing and Reinhard, 2019). Scheuble and Beauducel (2020) found a moderating effect of Machiavellianism on MFNs for female witnesses: The difference between MFN amplitudes of deceptive compared to honest responses was smaller for women scoring higher on Machiavellianism. Likewise, Panasiti et al. (2014) reported a lower BP before lying for participants higher in Machiavellianism. To sum up people high in Machiavellianism seem to have less scruple to lie, which could go along with a different cognitive processing of lying. Hence, additionally to replicating Johnson et al. (2008), we elucidated moderating effects of Machiavellianism.

## *Aims and Hypotheses*

Lying should be accompanied by more response conflicts and require more cognitive control processes, resulting in a) larger MFN and b) attenuated LPC amplitudes for lies compared to honest responses. Attitudes shape many behaviors and strategic monitoring can help to act in line with one’s lies. Accordingly, we expected c) attenuated PRP amplitudes for lies compared to honest responses. Lying about negatively valued items should be cognitively less challenging, since it can be perceived as a form of compliance and it is easier for people to recognize positive features for negatively valued themes. Therefore, we expected d) larger MFN and e) suppressed LPC amplitudes for deception of agree compared to deception of disagree items. Furthermore, we expected PRPs to be f) attenuated for agree compared to disagree items, since people tend to overestimate the population consensus for positively valued themes resulting in greater strategic monitoring in order to behave compliant to them. Additional to these effects, which were found by Johnson *et al.* (2008), we investigated moderating effects of Machiavellianism. Since people higher in Machiavellianism tend to lie more frequently, we expected that lying would be cognitively less strenuous for them resulting in g) smaller differences of MFN as well as h) LPC amplitudes between deceptive and honest responses. The associations of Machiavellianism with PRP amplitudes were explored.

# Method

## *Participants*

We collected data from 120 participants (see Supplement S2 for a description of the determination of the sample size). As in the study by Johnson et al. (2008), participants with less than 16 artifact free and correct trials in one of the analyzed categories (agree lie, agree honest, disagree lie, disagree honest) were excluded from analysis (n = 20). An additional participant had to be excluded because her response times of catch trials indicated that she likely reversed button assignments during the deception task^[[1]](#footnote-1)^ (cf. Johnson et al., 2008). A final sample of 99 participants (50 male; age: *M* = 21.28, *SD* = 2.80, range: 18-32 years) was available for analysis. Participants were right-handed, native German speakers, had normal or corrected-to-normal vision, did not take drugs or psychoactive medication and none of them had neurological, psychological or emotional disorders (cf. Johnson et al., 2008). All participants signed written informed consent before the examination and participated voluntarily. Psychology students (40%) got course credit for their participation. The study was performed in accordance with the revised Helsinki declaration **(2013)**. The local ethic board of the Institute of Psychology from the University of Bonn approved the study.

## *Measures*

The effects of different conceptions of Machiavellianism was controlled for by applying two different scales: an older German scale based on the questionnaire of Christie and Geis (1970) and a more recent scale (Henning and Six, 1977; Jones and Paulhus, 2014). Moreover, a total scale was formed as an equally weighted aggregate of the two scales. The internal consistency of the scale by Henning and Six (1977) was acceptable (α = .77), just like the internal consistency of the scale by Jones and Paulhus (α = .75). The internal consistency of the aggregate of both scales was moderate (α = .84).

The attitude questionnaire by Johnson et al. (2008) consists of items referring to religious, political and moral themes, well known people, and preferences. Some items are difficult to understand for German participants because they are based on content that is scarcely known in the German culture. Therefore, we excluded 12 (e.g. LASAR requirements, MEDICARE, workfare) of the 118 items of Johnson et al. (2008). Furthermore, we added items that are relevant for Germans nowadays (e.g. AfD, Angela Merkel, veganism; see Supplement S1). The final questionnaire consisted of 183 items. Participants were instructed to indicate how much they agree with the items, how strongly their attitudes are held, and how important their attitudes are. Responses were given on scales from one (extremely unfavorable/disagree; not at all strongly; not at all important) to seven (extremely favorable/agree; extremely strongly; extremely important). For the deception task we chose for each participant 26-30 items with the highest agreement (rated as 7, 6) as well as disagreement ratings (rated as 1, 2), selecting at first items that were also highly rated on the strength scale (Johnson et al., 2008). Johnson et al. (2008) also included 26-30 neutral items for each participant (rated as 4, 3 or 5 in the agreeableness scale). However, since people could have either ambivalent or no feelings towards them, they were not analyzed (Johnson et al., 2008). As the exclusion of these items might alter the cognitive processes during the task, we also included them into the deception task. Participants completed the attitude questionnaire once again after the deception task. This gave us the opportunity to control for changes in the attitudes.

## *Deception task*

We contacted Johnson, who kindly gave us additional information about the study (e.g. instructions of the deception task, items of the attitude questionnaire). All instructions and procedures were kept as close as possible to the original study. Participants saw stimuli on a computer screen and reacted to them by pressing either the left or the right of two buttons. During the deception task participants were instructed to indicate whether they agree or disagree with the items selected from the attitude questionnaire (Johnson et al., 2008). For neutral items, participants were instructed to indicate their tendency. During one block participants were instructed to respond honestly and in another block they were instructed to lie about their attitudes (Johnson et al., 2008). Each block consisted of 108 trials split into 18 catch trails and 90 trials with attitude items (Johnson et al., 2008). For catch trials, the words of the button assignments (“Agree” and “Disagree”) were presented on the screen and participants should respond to them with the corresponding button press, meaning, for example, pressing the button that stood for “I agree with the item” when the word “Agree” appeared (Johnson et al., 2008). Catch trials were included to ensure that participants did not simplify the task by swapping the meaning of the response buttons when they had to lie. The order of the two parts and the assignment of response buttons were counterbalanced across participants (Johnson et al., 2008). As in Johnson et al. (2008), the stimulus word was shown for 400 ms followed by an inter stimulus interval with a randomized duration of 2450-2850 ms (Figure 1). Participants could react to the stimulus word immediately or during the subsequent inter stimulus interval.

---Figure 1---

## *Procedure*

Participants completed the attitude questionnaire around one week (*M* = 6.87, *SD* = 1.07) before the EEG examination (cf. Johnson *et al.*, 2008). The EEG examination took part in a sound attenuated, electrically shielded, and well-lit room. Participants sat about 95 cm away from a 19´´ flat screen. The deception task was presented through Presentation V20.1 (Neurobehavioral Systems). It began with a simple orthographic task, so that participants could get to know the trial sequence (Johnson *et al.*, 2008). As in Johnson’s study, participants should discriminate two phonologically similar words: *Hafen* (haven) and *Hafer* (oat). Subsequently, participants completed the deception task.

## *EEG Recording and Quantification*

The EEG was recorded by an ActiveTwo BioSemi system (BioSemi, Amsterdam, Netherlands). Sixty-four active Ag/AgCl scalp electrodes were placed according to the extended 10/20 system (Jasper, 1958). Two additional electrodes were located at the pre-auricular sides. An electrooculogram (EOG) was formed by one electrode located at the middle of the forehead (FP1) and one electrode placed 2 cm below the outer canthus of the left eye (Johnson *et al.*, 2008). As per Biosemi design the Common Mode Sense active electrode and the Driven Right Leg passive electrode served as ground electrodes. Signals were digitized using ActiView software (BioSemi). The EEG was recorded with a sampling rate of 128 Hz, which is close to the sampling rate of 100 Hz used in Johnson *et al.* (2008). Offline analyses were performed with EEGLab (version 12.0.2.6b; Delorme and Makeig, 2004), based on MATLAB 7.14.739 (The MathWorks, Natick, MA). Data were re-referenced to averaged pre-auricular sides, filtered applying a 0.01-35 Hz band-pass filter, and segmented into epochs ranging from 1150 ms before until 300 ms after the response (baseline: -1150 to -1000 ms; Johnson *et al.*, 2008). Eye movements were removed by excluding epochs in which the signal of the EOG during any eight consecutive sampling points of the epoch exceeded 50 μV (cf. Johnson *et al.*, 2008). ERP components were quantified as mean amplitudes. MFNs were measured between 10 and 80 ms and PRPs between -100 and 0 ms at Fz, FC1, FC2, and Cz (Johnson *et al.*, 2008). LPCs were measured between -100 and +100 ms at P3, Pz, P4, CP1, and CP2 (Johnson *et al.*, 2008). Grand averages of fronto-central and parietal-central electrodes are depicted in Figures 2 and 3.

---Figure 2 and 3---

## *Statistical analysis*

Conventional statistical analyses were conducted with IBM SPSS (Version 24). Percentage of correct responses and reaction times served as behavioral data. For the percentage of correct responses, responses to attitude items that corresponded to the instructions (pressing the agree/disagree button for items one agreed with in the honest/deceptive block) were compared to those that did not correspond to the instructions (pressing the disagree/agree button for items one agreed with in the honest/deceptive block and missing responses). Repeated measures ANOVAs were computed for behavioral data and amplitude data for the LPC, MFN, and PRP ERP components with Response (honest vs. deception) and Valence (agree vs. disagree) as within subject factors (Johnson *et al.*, 2008). The ANOVAs of ERP data also included Electrode position as a within subject factor (Johnson *et al.*, 2008). To test whether LPC and MFN amplitudes differed among deceptive responses for agree and disagree items (hypothesis d and e), repeated measures ANOVAs were conducted including only deceptive responses (Johnson *et al.*, 2008). Separate repeated measures ANCOVAs were computed that included additionally the mean centered Machiavellianism scores as a covariate and Response × Machiavellianism as an interaction term. A description of the calculation of change scores for the attitude ratings are given in Supplement S3. Effects of violations of the sphericity assumption were corrected by means of Greenhouse-Geisser epsilon for the degrees of freedom and partial eta² is reported as an effect size (Johnson *et al.*, 2008). Only two-tailed levels of statistical significance are reported. Furthermore, we calculated Bayes factors using JASP (Version 0.10.0.0; JASP Team; 2018) because -in contrast to frequentist statistics- Bayes factors also allow for an interpretation of null results. We report BF_10_, which represents a ratio of the likelihood of our data under assumption of the alternative hypothesis and the likelihood of our data under assumption of the null hypothesis. For instance, a Bayes factor of 10.00 indicates that the data is 10 times more likely to occur under the alternative than under the null hypothesis. According to Jeffreys (1961) and Lee and Wagenmakers (2013), a Bayes factor of at least 3 indicates evidence for the alternative hypothesis and a Bayes factor of at least 100 indicates decisive evidence for the alternative hypothesis. A Bayes factor smaller than 1/3 indicates evidence for the null hypothesis. JASP does not calculate Bayes factors of single interaction terms. Therefore, Bayes factors of interaction terms were obtained by computing the differences between the ERP components for truthful and deceptive responses and using them as the dependent variable in the repeated measures Bayesian ANOVAs. The calculated Bayes factors of the main effects of these ANOVAs represent the interaction terms with the Response variable (truthful vs. deceptive responses).

We additionally conducted repeated measures ANOVAs of ERP components considering the data of all participants (without exclusion criteria). Categorization in significant results were the same as for the following reported analyses (see Supplement S4). Furthermore, additional Bootstrap analyses served to calculate the accuracy of detecting deception through patterns of LPC, MFN, and PRP amplitudes (cf. Rosenfeld *et al.*, 1991; Olson *et al.*, 2018). Methods and results of them can be found in Supplement S5 and S6.

# Results

## Ratings for agree and disagree items

The descriptive statistics for agree, disagree, and neutral items of the deception task from the present study and the study by Johnson *et al.* (2008) are summarized in Table 1. The evaluation, strength, and importance ratings are comparable to those of Johnson *et al.* (2008). Agree items were evaluated significantly more positive than disagree items, *t*(98) = 128.08, *p*< .001. Agreement and disagreement in topics differed in strength and importance with higher ratings for disagree items, *t*_strength_(98) = -4.11, *p*_strength_< .001; *t*_importance_(98) = -3.62, *p*_importance_< .001. The differences in the corresponding means were similar, although a bit smaller, for the present study compared to the study by Johnson *et al.* (2008).

---Table 1---

## Behavioral Data

The percentage of correct responses was higher for honest (*M* = 95.83%, *SE* = 0.37) compared to deceptive responses (*M* = 90.49%, *SE* = 0.59), *F*(1, 98) = 103.19, *p* < .001, *η_p_*^2^ = .51, BF_10_= 1.06e+20. Neither the main effect of Valence nor the interaction of Response × Valence were significant for the percentage of correct responses (all *ps* > .14, BF_10, Valence_= 0.14, BF_10, Response x Valence_= 0.43).

The repeated measures ANOVAs of response times revealed that responses were slower for deceptive than for honest responses (Table 2), *F*(1, 98) = 241.46, *p* < .001, *η_p_*^2^ = .71, BF_10_ = 3.43e+65. Furthermore, responses were slower for disagree than for agree items (Table 2), *F*(1, 98) = 18.79, *p* < .001, *η_p_*^2^ = .16. Yet, the corresponding Bayes factor suggested no evidence for the alternative hypothesis, BF_10_ = 0.38. The Response × Valence interaction was significant, *F*(1, 98) = 15.77, *p* < .001, *η_p_*^2^ = .14, BF_10_ = 143.81. The main effect of Valence was significant for truthful but not for deceptive responses, *F_truthful_*(1, 98) = 41.39, *p_truthful_* < .001, *η_p_*^2^*_truthful_* = .30, BF_10,_ *_truthful_* = 1.86e+6; *F_deception_*(1, 98) = 0.66, *p_deception_* = .42, *η_p_*^2^*_deception_* = .01, BF_10,_ *_deception_* = 0.20.

Repeated measures ANCOVAs that additionally considered Machiavellianism scores yielded no significant interactions between Response (honest vs. deceptive response) and Machiavellianism neither for response times nor for percentage of correct responses (all *ps* > .68, BF_10_ between 0.21 and 0.38).

---Table 2---

## MFN amplitudes

Repeated measures ANOVAs yielded a significant effect of Electrode Position, *F*(1.71, 167.07) = 30.08, *p* < .001, *η_p_*^2^ = .24, BF_10_ = 1.09e+6. Simple Contrasts revealed that MFN amplitudes were larger at Fz (*M* = -0.98 µV, *SE* = 0.45), than at FC1 (*M* = -0.51 µV, *SE*= 0.46; *F*(1, 98) = 5.02, *p* < .05, *η_p_*^2^ = .05), FC2 (*M* =-0.03 µV, *SE* = 0.47; *F*(1, 98) = 20.57, *p* < .001, *η_p_*^2^ = .17), and Cz (*M* = 1.49 µV, *SE* = 0.50; *F*(1, 98) = 39.09, *p* < .001, *η_p_*^2^ = .29). In accordance with hypothesis a, MFN amplitudes were larger for deceptive than for honest responses (Figure 4A), *F*(1, 98) = 28.10, *p* < .001, *η_p_*^2^ = .22. The corresponding Bayes factor suggested decisive evidence for hypothesis a, BF_10_ = 8.12e+18. Moreover, a significant main effect of Valence revealed that larger MFN amplitudes occurred for agree than for disagree items (Figure 4B), *F*(1, 98) = 6.98, *p* < .01, *η_p_*^2^ = .07, BF_10_ = 3,139.63. The Response × Valence interaction was not significant, *F*(1, 98) = 2.74, *p* = .10, *η_p_*^2^ = .03, although the Bayes factor provided evidence against the null hypothesis, BF_10_ = 95.17. Corresponding with hypothesis d, the Valence effect was significant for lies (Figure 4C), *F*(1, 98) = 6.70, *p* < .05, *η_p_*^2^= .06, BF_10_ = 305,984.01, but not for honest responses, *F*(1, 98) = 0.52, *p* = .47, *η_p_*^2^ = .01, BF_10_ = 0.22.

---Figure 4---

## PRP amplitudes

A significant main effect of Electrode Position occurred for PRP amplitudes, *F*(1.76, 172.41) = 22.74, *p* < .001, *η_p_*^2^ = .19, BF_10_ = 2,898.53. PRP amplitudes were smaller at Fz (*M* = 0.98 µV, *SE* = 0.39) compared to FC2 (*M* = 1.56 µV, *SE* = 0.42; *F*(1, 98) = 8.54, *p* < .01, *η_p_*^2^ = .08) and Cz (*M* = 2.89 µV, *SE* = 0.47; *F*(1, 98) = 28.74, *p* < .001, *η_p_*^2^ = .23). As expected in hypothesis c, PRP amplitudes were suppressed for deceptive compared to honest responses (Figure 5A), *F*(1, 98) = 49.12, *p*< .001, *η_p_*^2^ = .33, which was strongly supported by the Bayes factor, BF_10_= 1.85e+33. Furthermore, the main effect of Valence was significant (Figure 5B), *F*(1, 98) = 12.02, *p*< .001, *η_p_*^2^ = .11, BF_10_ = 1.57e+6. Corresponding with hypothesis f, PRP amplitudes were smaller for agree than for disagree items. The interaction of Response × Valence was not significant, *F*(1, 98) = 1.51, *p* = .22, *η_p_*^2^ = .02, whereas the corresponding Bayes factor suggested some evidence for the alternative hypothesis, BF_10_ = 3.60.

---Figure 5---

## LPC amplitudes

LPC amplitudes differed significantly depending on Electrode Position, *F*(2.64, 258.28) = 14.69, *p*< .001, *η_p_*^2^ = .13. LPC amplitudes were larger at Pz (*M* = 4.88 µV, *SE* = 0.47) than at P3 (*M* = 4.58 µV, *SE* = 0.44; *F*(1, 98) = 4.56, *p* < .05, *η_p_*^2^ = .04), P4 (*M* = 4.16 µV, *SE* = 0.43; *F*(1, 98) = 17.19, *p* < .001, *η_p_*^2^ = .15), CP1 (*M* = 3.88 µV, *SE* = 0.47; *F*(1, 98) = 30.88, *p*< .001, *η_p_*^2^ = .24), and CP2 (*M* = 3.66 µV, *SE* = 0.42; *F*(1, 98) = 63.85, *p* < .001, *η_p_*^2^ = .40). Yet, the Bayes factor of the main effect Electrode Position was inconclusive, BF_10_= 1.52. LPC amplitudes were smaller for deceptive compared to honest responses (Figure 6A), *F*(1, 98) = 50.16, *p*< .001, *η_p_*^2^ = .34, and also the corresponding Bayes factor suggested decisive evidence for hypothesis b, BF_10_ = 5.14e+67. Likewise, LPC amplitudes differed depending on the Valence of the items (Figure 6B), *F*(1, 98) = 8.55, *p* < .01, *η_p_*^2^ = .08, BF_10_ = 20,518.15. LPC amplitudes were smaller for agree compared to disagree items. The Response × Valence interaction was not significant, *F*(1, 98) = 2.08, *p* = .15, *η_p_*^2^ = .02. Yet, the corresponding Bayes factor indicated evidence for the alternative hypothesis, BF_10_ = 50.29. The repeated measures ANOVAs including only deceptive responses revealed that LPC amplitudes were smaller for agree than for disagree items (Figure 6C), *F*(1, 98) = 9.29, *p* < .01, *η_p_*^2^ = .09, BF_10_ = 2.55e+10 (supporting hypothesis e). The Valence effect was not significant for truthful responses, *F*(1, 98) = 0.89, *p*= .35, *η_p_*^2^ = .01, BF_10_ = 0.92.

---Figure 6---

## Machiavellianism and changes in the attitude ratings

Analyses on MFN, LPC, and PRP amplitudes considering Machiavellianism scores revealed no significant interaction with the Machiavellianism scores of the different scales (all *ps* > .15, BF_10_ between 0.22 and 0.42; hypotheses g and h were not confirmed). Likewise, no significant interaction occurred between Response and changes in the attitude rating (all *ps* >.13, BF_10_ between 0.32 and 0.60). Significant results of Response and Valence remained significant when considering Machiavellianism or changes in the attitudes.

# Discussion

In the present study we found strong support that deception about attitudes relies on additional cognitive control processes used to monitor and resolve response conflicts in comparison to being honest. We replicated the following results of Johnson *et al.* (2008): MFN amplitudes were larger for lies indicating that lying was accompanied by stronger response conflicts than telling the truth (hypothesis a). LPC amplitudes were smaller for lies suggesting that additional cognitive resources were required for responding deceptively (hypothesis b). Lying about attitudes triggered strategic monitoring as indicated by suppressed PRP amplitudes for lies compared to truthful responses (hypothesis c). Cognitive processes also differed depending on the valence of the attitudes. Lying about positively valued items was accompanied by more intense response conflicts and was cognitively more challenging than lying about negatively valued items. MFN amplitudes were larger and LPC amplitudes suppressed for lies about items one agrees with than for lies about topics one disagrees with (hypotheses d and e). PRPs were suppressed for agree compared to disagree items revealing that ratings about positively viewed topics trigger greater strategic monitoring than ratings about negatively viewed topics (hypothesis f). In addition to replicating Johnson *et al.* (2008), we found that cognitive processes indicated by MFN, PRP, and LPC amplitudes during lying were not moderated by Machiavellianism (hypotheses g and h not confirmed).

Even though our effect sizes were not as large as in the study by Johnson *et al.* (2008), they still represent clear evidence for the alternative hypothesis, as indicated by Bayes factors (cf. Jeffreys, 1961). Corresponding to the ERP results, reactions were slower and participants made more mistakes when they lied about their attitudes. The results are in line with previous reaction time and ERP studies implying that lying is accompanied by greater response inhibition and that executive control processes are needed to handle the greater cognitive demands of lies (Johnson *et al.*, 2003, 2004, 2005, 2008; Vendemia and Buzan, 2005; Suchotzki *et al.*, 2017). Furthermore, they reveal that MFN, LPC, and PRP components are reliable indicators of the cognitive processes of deception even beyond the context of stimulus recognition.

However, bootstrap analyses revealed that, based on intra-individual patterns of the ERP components and current categorization criteria, only a small amount of people could be categorized as lying (Supplement S5 and S6) and that further research is needed to reach this goal. In accordance with conclusions from other studies, these results seem to indicate that there is, at least as of now, no Pinocchio’s nose (Fischbach and Fischbach, 2005; Volbert and Banse, 2014). The goal of the present study, as in the original study by Johnson *et al.* (2008), was to uncover the underlying cognitive processes of deception. Both studies revealed not one but multiple ERP markers of the cognitive processes used during deception. These markers could be combined with other ERPs as well as behavioral markers, such as differences in the speed and accuracy of truthful and deceptive responses, in a deception detection algorithm (cf. Johnson *et al.*, 2014). In future studies concentrating on the detection of deception, participants that only tell the truth could be compared to lying participants, making it possible to adjust the criteria for categorizing lies by considering the rate of false positives (honest participants categorized as liars) and false negatives (lying participants categorized as being honest) and finding the best way to combine ERP and behavioral markers of deception. The possibility to base such an algorithm on a variety of deception markers has the potential to combat countermeasures, since deception can be classified based on all or a subset of these markers (Johnson *et al.*, 2014). It is probably very difficult to willingly manipulate all of these ERP components, especially since they occur at different, brief periods of time, index different cognitive processes, and are generated in varying brain areas (Johnson *et al.*, 2014).

Machiavellianism neither modulated differences in ERP components nor in response times nor in the correctness of responses suggesting that deception was not cognitively less strenuous for individuals higher in Machiavellianism. At least in this deception setting, when rating attitudes, individuals higher in Machiavellianism do not seem to experience fewer conflicts during lying. Since persons higher in Machiavellianism deceive more frequently in their everyday life, our finding is in line and expands the result of the study by Johnson *et al.* (2005) that practice did neither moderate behavioral nor MFN, PRP, and LPC components during deception about the memorization of words. The non-significant Machiavellianism effects may indicate that at least for lies about attitudes the components are not so much affected by the moral or ethical standards of the individual, but rather by the resources required for cognitive processing of lies (Johnson *et al.*, 1986, 1988). Moreover, we found that changes in the attitude ratings (before vs. after the deception task) did not result in smaller differences of MFN, PRP, and LPC amplitudes during the deception task. Altogether additional analyses regarding Machiavellianism, changes in attitude ratings, as well as additional statistical tests with Bayes factors underline the stability of the ERP effects.

## Limitations and future directions

As this was the first replication of Johnson *et al.* (2008), we conducted our study as similar as possible to the original study. We could replicate their main ERP findings with a sample from another country. The same pattern of MFN, LPC, and PRP results were obtained for German as for US residents. Yet, as in the original study, participants were from a western, industrialized country. It would be interesting to analyze data from individuals beyond the western culture. Moreover, as in Johnson *et al.* (2008) our study sample comprised mainly students. We cannot rule out that a moderating effect of Machiavellianism occurs for a sample with more extreme values in Machiavellianism, e.g. for prisoners or a clinical sample. Furthermore, it remains for future studies to find out whether such a moderation can be found in other deception settings, for example, when lying goes along with more positive consequences than truth telling.

## Conclusion

We could replicate the main ERP results of the study by Johnson *et al.* (2008). Lying was accompanied by larger MFN, suppressed LPC, and suppressed PRP amplitudes indicating that it was accompanied by more conflicts and was cognitively more challenging than truth telling. Hence, the findings of Johnson *et al.* (2008) could be generalized to a German-speaking sample. Our findings indicate that it is promising to elucidate cognitive processes during deception through ERP components in non-recognition contexts. Cognitive processes, resolving response conflicts, handling the greater mental workload and applying strategic monitoring, seem to be basic when lying about attitudes, since the same patterns of LPC, MFN, and PRP results were repeatedly obtained when analyzing deception of attitudes. Moreover, the patterns of these ERP components were stable for changes in attitude ratings and across individual differences in Machiavellianism.

Words in text: 5929

**Conflict of Interest**

The authors declare no conflict of interest.

**Acknowledgements**

We are grateful to Niklas Niedermeier, Sophie Schormann, Sabine Sedlaczek, Leona Wahnschaffe, and Leon Dehnhardt for their help during data collection. We thank Benjamin Cabrera for helpful advice during programming.

# References

Allen, J.J., Iacono, W.G. (1997). A comparison of methods for the analysis of event-related potentials in deception detection. *Psychophysiology*, **34**, 234–40. doi: 10.1111/j.1469-8986.1997.tb02137.x.

Aron, A.R., Robbins, T.W., Poldrack, R.A. (2004). Inhibition and the right inferior frontal cortex. *Trends in Cognitive Sciences*, **8**, 170–77. doi: 10.1016/j.tics.2004.02.010.

Azizli, N., Atkinson, B.E., Baughman, H.M., Chin, K., Vernon, P.A., Harris, E., et al. (2016). Lies and crimes: Dark Triad, misconduct, and high-stakes deception. *Personality and Individual Differences*, **89**, 34–39. doi: 10.1016/j.paid.2015.09.034.

Beauducel, A., Brocke, B., Leue, A. (2006). Energetical bases of extraversion: Effort, arousal, EEG, and performance. *International Journal of Psychophysiology*, **62**, 212–23. doi: 10.1016/j.ijpsycho.2005.12.001.

Botvinick, M.M. (2007). Conflict monitoring and decision making: Reconciling two perspectives on anterior cingulate function. *Cognitive, Affective, & Behavioral Neuroscience*, **7**, 356–66. doi: 10.3758/CABN.7.4.356.

Cohen, J. (1988). *Statistical Power Analysis for the Behavioral Sciences*. Hillsdale, NJ: Lawrence Erlbaum Associates.

Dong, G., Wu, H., Lu, Q. (2010). Attempting to hide our real thoughts: Electrophysiological evidence from truthful and deceptive responses during evaluation. *Neuroscience Letters*, **479**, 1–5. doi: 10.1016/j.neulet.2010.05.014.

Fazio, R.H., Pietri, E.S., Rocklage, M.D., Shook, N.J. (2015). Positive versus negative valence: Asymmetries in attitude formation and generalization as fundamental individual differences. In: J. M. Olson, M. P. Zanna (eds). *Advances in Experimental Social Psychology*. Burlington, NJ: Academic Press, p. 97–146. doi: 10.1016/bs.aesp.2014.09.002.

Fischbach, R.L., Fischbach, G.D. (2005). The brain doesn’t lie. *American Journal of Bioethics*, **5**, 54–55. doi: 10.1080/15265160590960979.

Gehring, W.J., Willoughby, A.R. (2004). Are all medial frontal negativities created equal? Toward a richer empirical basis for theories of action monitoring. In: M. Ullsperger, M. Falkenstein (eds). *Errors, conflicts, and the brain: Current opinions on performance monitoring*. Leipzig: Max Planck Institute for Human Cognitive and Brain Sciences, p. 14–20.

Gershoff, A.D., Mukherjee, A., Mukhopadhyay, A. (2008). What’s not to like? Preference asymmetry in the false consensus effect. *Journal of Consumer Research*, **35**, 119–25. doi: 10.1086/524416.

Gibbons, H., Schnuerch, R., Wittinghofer, C., Armbrecht, A.S., Stahl, J. (2018). Detection of deception: Event-related potential markers of attention and cognitive control during intentional false responses. *Psychophysiology*, **55**, 1–22. doi: 10.1111/psyp.13047.

Gombos, V.A. (2006). The cognition of deception: The role of executive processes in producing lies. *Genetic, Social, and General Psychology Monographs*, **132**, 197–214. doi: 10.3200/MONO.132.3.197-214.

Gozna, L.F., Vrij, A., Bull, R. (2001). The impact of individual differences on perceptions of lying in everyday life and in a high stake situation. *Personality and Individual Differences*, **31**, 1203–16. doi: 10.1016/S0191-8869(00)00219-1.

Henning, H.J., Six, B. (1977). Konstruktion einer Machiavellismus-Skala [Construction of a Machiavellianism Scale]. *Zeitschrift für Sozialpsychologie*, **8**, 185–98.

Jasper, H.H. (1958). The ten-twenty electrode system of the International Federation. *Electroencephalography and Clinical Neurophysiology*, **10**, 371–75.

Jeffreys, H. (1961). *Theory of probability*. 3rd ed. Oxford: Oxford University Press.

Johnson Jr., R. (2014). The neural basis of deception and credibility assessment: A cognitive neuroscience perspective. In: *Credibility Assessment: Scientific Research and Applications*. Oxford: Elsevier, p. 217–300. doi: 10.1016/B978-0-12-394433-7.00006-3.

Johnson Jr., R. (1988). The amplitude of the P300 component of the event-related potential: Review and synthesis. In: P. Ackles, J.R. Jennings, M. G. H. Coles (eds). *Advances in Psychophysiology*. Greenwich, CT: JAI Press, p. 69-137.

Johnson Jr., R. (1986). A triarchic model of P300 amplitude. *Psychophysiology*, **23**, 367–84 . doi: 10.1111/j.1469-8986.1986.tb00649.x.

Johnson Jr., R., Barnhardt, J., Zhu, J. (2005). Differential effects of practice on the executive processes used for truthful and deceptive responses: An event-related brain potential study. *Cognitive Brain Research*, **24**, 386–404. doi: 10.1016/j.cogbrainres.2005.02.011.

Johnson Jr., R., Barnhardt, J., Zhu, J. (2004). The contribution of executive processes to deceptive responding. *Neuropsychologia*, **42**, 878–901. doi: 10.1016/j.neuropsychologia.2003.12.005.

Johnson Jr., R., Barnhardt, J., Zhu, J. (2003). The deceptive response: Effects of response conflict and strategic monitoring on the late positive component and episodic memory-related brain activity. *Biological Psychology*, **64**, 217–53. doi: 10.1016/j.biopsycho.2003.07.006.

Johnson Jr., R., Henkell, H., Simon, E., Zhu, J. (2008). The self in conflict: The role of executive processes during truthful and deceptive responses about attitudes. *NeuroImage*, **39**, 469–82. doi: 10.1016/j.neuroimage.2007.08.032.

Jones, D.N., Paulhus, D.L. (2014). Introducing the short dark triad (SD3): A brief measure of dark personality traits. *Assessment*, **21**, 28–41. doi: 10.1177/1073191113514105.

Kashy, D.A., DePaulo, B.M. (1996). Who lies? *Journal of Personality and Social Psychology*, **70**, 1037–51. doi: 10.1037/0022-3514.70.5.1037.

Kireev, M. V, Pakhomov, S. V, Medvedev, S. V (2008). Cerebral mechanisms of error detection during deceptive responses in the normal state and under the influence of alcohol. *Human Physiology*, **34**, 141–49. doi: 10.1134/S0362119708020023.

Lee, M.D., Wagenmakers, E.-J. (2013). *Bayesian cognitive modeling: A practical course*. C. U. Press (ed).

Leue, A., Beauducel, A. (2019). A meta-analysis of the P3 amplitude in tasks requiring deception in legal and social contexts. *Brain and Cognition*, **135**. doi: 10.1016/j.bandc.2019.05.002.

Leue, A., Lange, S., Beauducel, A. (2012). “Have you ever seen this face?” - Individual differences and event-related potentials during deception. *Frontiers in Psychology*, **3**, 1–13. doi: 10.3389/fpsyg.2012.00570.

Meek, S.W., Phillips, M.C., Boswell, C.P., Vendemia, J.M.C. (2013). Deception and the misinformation effect: An event-related potential study. *International Journal of Psychophysiology*, **87**, 81–87. doi: 10.1016/j.ijpsycho.2012.11.004.

Miller, M.W., Rietschel, J.C., McDonald, C.G., Hatfield, B.D. (2011). A novel approach to the physiological measurement of mental workload. *International Journal of Psychophysiology*, **80**, 75–78. doi: 10.1016/j.ijpsycho.2011.02.003.

Nieuwenhuis, S., Holroyd, C.B., Mol, N., Coles, M.G.H. (2004). Reinforcement-related brain potentials from medial frontal cortex: Origins and functional significance. *Neuroscience and Biobehavioral Reviews*, **28**, 441–48. doi: 10.1016/j.neubiorev.2004.05.003.

Olson, J., Rosenfeld, J.P., Kim, T., Perrault, E. (2018). The effect of countermeasures against the reaction time based concealed information test on the P300 index of knowledge recognition: A combined RT and P300-based test. *International Journal of Psychophysiology*, **134**, 9–14. doi: 10.1016/j.ijpsycho.2018.10.003.

Open Science Collaboration (2015). Estimating the reproducibility of psychological science. *Science*, **349**, 943-aac4716-8. doi: 10.1126/science.aac4716.

Palmer, B., Nasman, V.T., Wilson, G.F. (1994). Task decision difficulty: effects on ERPs in a same-different letter classification task. *Biological Psychology*, **38**, 199–214. doi: 10.1016/0301-0511(94)90039-6.

Panasiti, M.S., Pavone, E.F., Mancini, A., Merla, A., Grisoni, L., Aglioti, S.M. (2014). The motor cost of telling lies: Electrocortical signatures and personality foundations of spontaneous deception. *Social Neuroscience*, **9**, 573–89. doi: 10.1080/17470919.2014.934394.

Pfister, R., Foerster, A., Kunde, W. (2014). Pants on fire: The electrophysiological signature of telling a lie. *Social Neuroscience*, **9**, 562–72. doi: 10.1080/17470919.2014.934392.

Polich, J. (2007). Updating P300: An integrative theory of P3a and P3b. *Clinical Neurophysiology*, **118**, 2128–48. doi: 10.1016/0301-0511(95)05130-9.

Polich, J., Kok, A. (1995). Cognitive and biological determinants of P300: An integrative review. *Biological Psychology*, **41**, 103–46. doi: 10.1016/0301-0511(95)05130-9.

Rosenfeld, J.P., Angell, A., Johnson, M., Qian, J.-H. (1991). An ERP-based, control-question lie detector analog: Algorithms for discriminating effects within individuals’ average waveforms. *Psychophysiology*, **28**, 319–35. doi: https://doi.org/10.1111/j.1469-8986.1991.tb02202.x.

Rosenfeld, J.P., Hu, X., Labkovsky, E., Meixner, J., Winograd, M.R. (2013). Review of recent studies and issues regarding the P300-based complex trial protocol for detection of concealed information. *International Journal of Psychophysiology*, **90**, 118–34. doi: 10.1016/j.ijpsycho.2013.08.012.

Scheuble, V., Beauducel, A. (2020). Individual differences in ERPs during deception: Observing vs. demonstrating behavior leading to a small social conflict. *Biological Psychology,* **150**, 1-11*.* doi: 10.1016/j.biopsycho.2019.107830.

Sip, K.E., Roepstorff, A., McGregor, W., Frith, C.D. (2008). Detecting deception: the scope and limits. *Trends in Cognitive Sciences*, **12**, 48–53. doi: 10.1016/j.tics.2007.11.008.

Suchotzki, K., Crombez, G., Smulders, F.T.Y., Meijer, E., Verschuere, B. (2015). The cognitive mechanisms underlying deception: An event-related potential study. *International Journal of Psychophysiology*, **95**, 395–405. doi: 10.1016/j.ijpsycho.2015.01.010.

Suchotzki, K., Verschuere, B., Van Bockstaele, B., Ben-Shakhar, G., Crombez, G. (2017). Lying takes time: A meta-analysis on reaction time measures of deception. *Psychological Bulletin*, **143**, 428–53. doi: 10.1037/bul0000087.

Tu, S., Li, H., Jou, J., Zhang, Q., Wang, T., Yu, C., et al. (2009). An event-related potential study of deception to self preferences. *Brain Research*, **1247**, 142–48. doi: 10.1016/j.brainres.2008.09.090.

Vendemia, J.M.C., Buzan, R.F. (2005). Neural mechanisms of deception and response congruity in a visual two-stimulus paradigm with motor response. *Polygraph*, **34**, 24–46.

Volbert, R., Banse, R. (2014). Deception detection: How can psychological research assist legal practice? *European Psychologist*, **19**, 159–61. doi: 10.1027/1016-9040/a000209.

Vrij, A., Fisher, R., Mann, S., Leal, S. (2006). Detecting deception by manipulating cognitive load. *Trends in Cognitive Sciences*, **10**, 141–42. doi: 10.1016/j.tics.2006.02.003.

Wissing, B.G., Reinhard, M.A. (2019). The dark triad and deception perceptions. *Frontiers in Psychology*, **10,** 1-7. doi: 10.3389/fpsyg.2019.01811.

Yong, E. (2012). Bad copy. *Nature*, **485**, 298–300. doi: 10.1038/485298a.

**Figure captions**

**Fig. 1.** Sequence of two trials of the deception task.

**Fig. 2.** Response-locked grand averages of fronto-central electrodes for lies as well as truthful responses for items participants agreed and disagreed to. Epochs spanned from 1150 ms before until 300 ms after the response. One tick at the x-axis stands for 100 ms and one tick at the y-axis for one µV.

**Fig. 3.** Response-locked grand averages of parietal-central electrodes for lies as well as truthful responses for items participants agreed and disagreed to. Epochs spanned from 1150 ms before until 300 ms after the response. One tick at the x-axis stands for 100 ms and one tick at the y-axis for one µV.

**Fig. 4.** Means and standard errors of MFN amplitudes representing A) the Response main effect (lies vs. truthful responses), B) the Valence effect (agree vs. disagree items) and C) the Valence effect for lies (lies for agree vs. lies for disagree items).

**Fig. 5.** Means and standard errors of PRP amplitudes representing A) the Response main effect (lies vs. truthful responses) and B) the Valence effect (agree vs. disagree items).

**Fig. 6.** Means and standard errors of LPC amplitudes representing A) the Response main effect (lies vs. truthful responses), B) the Valence effect (agree vs. disagree items), and C) the Valence effect for lies (lies for agree vs. lies for disagree items).

1. For catch trials participants had to indicate honestly the button assignments (see section “Deception task”). The cognitive demands for catch trials are therefore much lower than for deceptive responses. Yet, the mean response times of the excluded participant were higher for catch trials than for trials of attitude items requiring a deceptive response indicating that the task was not performed as instructed. [↑](#footnote-ref-1)
